# The underlying mechanisms by which Post-Traumatic Growth is associated with cardiovascular health in male UK military personnel: The ADVANCE cohort study

**DOI:** 10.1177/13591053241240196

**Published:** 2024-04-11

**Authors:** Daniel Dyball, Alexander N Bennett, Susie Schofield, Paul Cullinan, Christopher J Boos, Anthony MJ Bull, Sharon AM Stevelink, Nicola T Fear

**Affiliations:** 1 King’s College London, UK; 2 Defence Medical Rehabilitation Centre, UK; 3 Imperial College London, UK; 4 Bournemouth University, UK

**Keywords:** ADVANCE cohort study, Afghanistan, heart disease risk factors, military personnel, posttraumatic growth, psychological

## Abstract

Post-Traumatic Growth (PTG) is associated with good cardiovascular health, but the mechanisms of this are poorly understood. This cross-sectional analysis assessed whether factors of PTG (Appreciation of Life (AOL), New Possibilities (NP), Personal Strength (PS), Relating to Others (RTO) and Spiritual Change (SC)) are associated with cardiovascular health in a cohort of 1006 male UK military personnel (median age 34). The findings suggest AOL, PS and RTO are associated with better cardiovascular health through cardiometabolic effects (lower levels of triglycerides, and total cholesterol) and haemodynamic functioning (lower diastolic blood pressure), but not inflammation. However, NP and SC were associated with poorer cardiovascular health through cardiometabolic effects (lower levels of high-density lipoproteins and higher levels of total cholesterol) and AOL had a non-linear association with low-density lipoproteins. These findings suggest that the relationship between PTG and cardiovascular functioning is complex and in need of further scrutiny.

## Introduction

The understanding of disorders such as depression, anxiety, Post-Traumatic Stress Disorder (PTSD) and other negative responses to stress or trauma, along with interventions and treatments for these pathologies, has progressed considerably over the past century. However, a focus on mental illness limits our understanding of the full spectrum of mental health, both positive and negative, and their associations with physical health (Labarthe et al., 2016). Investigation into aspects of positive psychology has become more common in recent years, with more attention being paid to the full range of human experience, inclusive of psychological thriving rather than just pathology.

Post-Traumatic Growth (PTG) is one such aspect of psychological thriving which has received notable attention in the scientific community. PTG refers to beneficial psychological change following trauma. One of the most used measures of PTG, the PTG-Inventory, determines PTG through five factors: a greater Appreciation Of Life (AOL), seeing New Possibilities (NP) in one’s future, an increase in one’s perception of their own Personal Strength (PS), Relating To Others (RTO) better and positive Spiritual Change (SC) (Lee et al., 2010; Tedeschi and Calhoun, 1996). PTG has been shown to be prevalent amongst many trauma survivors, including those exposed to warzones and life-threatening illnesses (Barskova and Oesterreich, 2009; Dyball et al., 2023a; Mark et al., 2018). Importantly, research has shown that PTG can be elicited through psychological intervention, meaning those that experience negative reactions to traumatic events, such as PTSD, can be helped to build beneficial psychological changes alongside treating and minimising the symptoms of PTSD (Murphy et al., 2017).

The pathology of mental illness has been linked to physical illness. For example, PTSD has been shown to be associated with Cardiovascular Disease (CVD) through risk factors such as inflammation, cardiometabolic effects and haemodynamic functioning (Dyball et al., 2019, 2023b; Edmondson and von Känel, 2017). Positive psychology is theorised to affect these physiological dimensions through increases in restorative processes such as sleep, physical activity and diet, or functioning as a protective factor against deleterious functions such as stress/cortisol response (Boehm, 2021; Jobin et al., 2014; Lai et al., 2005). Positive psychological constructs, including optimism (a perspective that life ahead will be good), positive affect (the extent of which a person feels positive emotions), resilience (an ability to adapt positively to life conditions) and PTG, have been found to be associated with better cardiovascular health (Amonoo et al., 2021; Dubois et al., 2012; Rozanski et al., 2019; Sisto et al., 2019; Teixeira et al., 2018). In those with established CVD, positive psychological interventions including optimism training have been found to lower cortisol and inflammatory response (Dubois et al., 2012; Mohammadi et al., 2020; Nikrahan et al., 2016). In those without established CVD, PTG and positive psychological functioning have been found to be associated with positive lifestyle factors such as better physical fitness or smoking tobacco (Amonoo et al., 2021), and better cardiovascular risk profiles including better haemodynamic functioning (Hourani et al., 2020; Thege et al., 2015), cardiometabolic effects (Schiavon et al., 2020; Tran et al., 2011) and inflammation (Marteinsdottir et al., 2016). There remains a question as to whether measuring these positive psychological constructs and their association with physical health are actually just measuring the absence of negative constructs (e.g. depression, anxiety or PTSD) (Dubois et al., 2012). To the authors best knowledge, no studies have investigated the psychological mechanisms by which PTG might affect cardiovascular health indicators through investigation of the five factors of PTG.

The ArmeD serVices trAuma rehabilitatioN outComE (ADVANCE) study is a large cohort of UK military personnel, approximately half of which sustained a physical combat injury and half are a frequency-matched uninjured comparison group (Bennett et al., 2020). Using variable selection procedures including Bootstrap Inclusion Frequencies (BIF) and model averaging, novel statistical approaches to reduce bias in models through the inclusion/exclusion of pertinent variables (Heinze et al., 2018; Sauerbrei et al., 2020), a recent investigation using the ADVANCE cohort data found that PTSD symptom clusters were associated with cardiovascular health factors including cardiometabolic effects and haemodynamic functioning (Dyball et al., 2023b), but not inflammation (specifically inflammatory marker High-sensitivity C-Reactive Protein (HsCRP). PTG has also been investigated in this cohort, with 28% of the uninjured group and 36% of the injured group experiencing a large degree of PTG (Dyball et al., 2023a).

Based on the emerging scientific literature that suggests positive psychology is associated with better cardiovascular health, we hypothesise that PTG will be associated with better cardiovascular health indicators including inflammation, cardiometabolic effects and haemodynamic functioning. We aim to assess the relative importance of the factors of PTG in these associations via variable selection procedures and confirm results via linear regression modelling.

## Methods

### Participants

This study is secondary data analysis from the baseline data collection of the ADVANCE cohort study, a longitudinal investigation into the long-term health impact of sustaining a battlefield injury (Bennett et al., 2020). Participants consisted of 579 physically injured and 566 uninjured UK military personnel who deployed to Afghanistan. The uninjured group were frequency-matched to the injured group based on sex, age, rank, role on deployment, regiment and deployment era. There were only a very small number of female UK military combat casualties in Afghanistan. Due to this and physiological differences between males and females that would confound the primary hypotheses of ADVANCE, only male UK injured personnel were eligible for this cohort study.

### Procedure

Participants were assessed at the UK Defence Medical Rehabilitation Centre (DMRC) Headley Court (2015–2018) or Stanford Hall (2018–2020). The ADVANCE assessment day included a comprehensive health assessment including self-report questionnaires, a research nurse-led clinical interview, Vicorder assessment, bloodwork, and Dual-Energy X-ray Absorptiometry (DEXA). A measure of PTG was introduced to the study in 2018. From this point, participants completed the questionnaire as part of their ADVANCE assessment. Participants who attended prior to this date were invited to complete the questionnaire either online or via post.

### Ethics

The ADVANCE Study has full ethical approval from the UK Ministry of Defence Research Ethics Committee (MODREC; protocol No:357/PPE/12). All participants gave written informed consent and investigation was carried out in accordance with the 2013 version of the declaration of Helsinki.

### Materials

#### Post-traumatic growth

PTG was assessed using the Deployment-related Post-Traumatic Growth Inventory (DPTGI), a military deployment variant of the Post-Traumatic Growth Inventory (Dyball et al., 2022b, 2023a; Tedeschi and Calhoun, 1996). The stem question relates to ‘as a result of my deployments to Iraq or Afghanistan since 2002’:. The measure contains 21 items and scores range from 0 to 63. The five-factor scoring method includes: AOL (*n* = 3 items; score 0–9), NP (*n* = 5; score 0–15), PS (*n* = 4 items; score 0–12); RTO (*n* = 7 score 0–21) and SC (*n* = *2* items; score 0–6). Cronbach’s alpha for the measure was 0.94, with the factors ranging from 0.73 (SC) to 0.88 (RTO).

#### Anxiety

Anxiety was assessed using the Generalised Anxiety Disorder-7 (GAD), which contains seven items and scores ranging from 0 to 21 (Spitzer et al., 2006). Probable anxiety was defined as a score of ≥ 10.

#### Depression

Depression was assessed using the Patient Health Questionnaire-9 (PHQ), which contains nine items and scores ranging from 0 to 27 (Kroenke et al., 2001). Probable depression was defined as a score of ≥10.

#### Post-traumatic stress disorder

PTSD was assessed using the PTSD Clinical Checklist (PCL-C). The measure has 17 items and scores range from 17 to 83 (Blanchard et al., 1996). Probable PTSD was defined as a score of ≥ 50.

### Outcomes

Classification of normal clinical ranges for each cardiovascular outcome can be found in Supplemental materials 1.

### Inflammation

#### High-sensitivity C-reactive protein (HsCRP)

Venous blood sampling was conducted on-site and assayed at a local NHS laboratory. HsCRP was measured in mg/l, with a lower detection limit of 0.10 mg/l.

### Haemodynamic functioning

#### Vicorder assessment

Participants were laid in a supine position at a 30-degree angle for a Vicorder (Skidmore Medical, UK) assessment. After a 5-minute rest period, participants had assessments of diastolic and systolic blood pressure along with resting heart rate and pulse wave velocity. Measurements were taken three times by a research nurse in a temperature-controlled environment. Resting heart rate (Beats Per Minute (BPM)) and brachial systolic/diastolic blood pressure (millimetres of mercury (mmHg)) were taken during pulse wave analysis, assessed from the cuff of the left upper arm and left thigh. Pulse wave velocity (metres per second (m/s)) was assessed from the cuff of the left upper arm and neck. Mean values across the three readings were taken as per recommended guidance (Van Bortel et al., 2012), however if pulse wave velocity readings differed by ≥0.5 m/s, the median value was taken. Similarly, for blood pressure and heart rate readings, the median was taken if readings differed by 2.5x the median absolute deviation (Leys et al., 2013). Observations where all three readings were greater than 2.5x the median absolute deviation were removed from the analysis (the number of excluded observations ranged from 15 (resting heart rate) to 53 (pulse wave velocity)).

### Cardiometabolic effects

Venous blood sampling was undertaken in the morning of the participant’s appointment. Participants fasted for at least 8 hours prior to venous blood sampling, including no caffeine or alcohol. Blood plasma and serum samples were assayed at local NHS laboratories.

#### Blood glucose and insulin resistance

Fasting glycated haemoglobin (HbA1_C_) was measured in mmol/mol. Conversion to HbA1_C_ % was conducted for the purposes of estimating insulin resistance using the International Federation of Clinical Chemistry-National Glycohemoglobin Standardisation Programme equation (Braatvedt et al., 2012). Estimated Glucose Disposal Rate (eGDR) was used as an indicator of insulin resistance (Boos et al., 2022). Lower eGDR is reflective of greater insulin resistance and is measured in milligrammes/kilograms per minute (mg/kg/min). eGDR was calculated as: eGDR mg/kg/minute = 21.158−(0.09 × abdominal waist circumference (cm))−(3.407 × hypertension (yes = 1, no = 0))−(0.551 × HbA1_C_ %). Hypertension was defined as medication use with the indication of hypertension, or current high blood pressure defined as systolic blood pressure (≥140 mmHg) and diastolic blood pressure (≥90 mmHg).

#### Dyslipidaemia

Triglycerides, total cholesterol, High Density Lipoproteins (HDL), Low Density Lipoproteins (LDL) were assessed from blood samples in mmol/l. HDL refers to lipids that absorb other cholesterol for processing/recycling in the liver, and higher levels of HDL is associated with lower CVD risk (Nhlbi, 2001). LDL cholesterol refers to cholesterol that circulates in the body for depositing inside or artery walls/cell-repair. Higher levels of LDL are associated with greater CVD risk (Nhlbi, 2001). Data was transformed to mg/dl for the purposes of this study to increase interpretability (Rugge et al., 2012).

#### Obesity

Participants completed a full body DEXA (Vertec Horizon Discovery, UK) scan. Participants were laid in a supine position with neck and spine aligned to the centre of the DEXA table, legs apart with feet turned inwards. Visceral adipose tissue was measured in cm^2^.

### Confounders

#### Age

Age was measured at time of the ADVANCE assessment in years.

#### Combat injury

Combat injury details were collected from electronic medical records provided by the Ministry of Defence: Defence Statistics (Health) department and supplemented by self-report data collected during the clinical interview section. Combat injury was categorised as uninjured or injured (Boos et al., 2022).

#### Medication

Ethnicity was self-reported during the clinical interview. Answers were coded as ‘Asian’, ‘Black’, ‘Mixed’ or ‘White’. A binary code was derived from this data to represent ‘White’ and ‘Ethnic minorities (not including white minorities)’.

#### Medication

Self-reported current medication use was collected during the clinical interview. The Anatomical Therapeutic Chemical Classification Index (World Health Organisation Collaborating Centre for Drug Statistics Methodology, 2020) was used to code medications. Medications of interest for this current study included medications with a primarily cardiovascular or mental health effect including: anti-gout preparations; agents acting on the renin-angiotensin system; antihypertensives; calcium channel blockers; corticosteroids for systemic use; diuretics; drugs used in diabetes; immunosuppressants; lipid modifying agents; anabolic agents for systemic use; psychoanaleptics (drugs that produce a calming mental health effect) and psycholeptics (drugs that provide a stimulating mental health effect). Medication use was categorised as ‘not on medication of interest’ and ‘on medication of interest’.

#### Socioeconomic status

Socioeconomic status was categorised based on rank at time of sampling; junior non-commissioned officer rank (NATO OR2-OR4), senior non-commissioned officer rank (NATO OR5-OR9) and commissioned officer rank (NATO OF1-OF6) (Yoong et al., 1999).

### Data analysis

Data analysis was conducted using STATA 17.0. Confounders, based a-priori on the literature, included age, combat injury, medication of interest, socioeconomic status and ethnicity (Boos et al., 2022; Clark et al., 2009; Gasevic et al., 2015; Kurian and Cardarelli, 2007; Lakatta, 2002; Mark et al., 2018; Mwebe and Roberts, 2019). Regression diagnostics were completed on linear regression models including all centred PTG factors and each outcome to assess normality of residuals, influential observations (Cook’s D) and multicollinearity (variance inflation factor). Variables were transformed if residual normality was not achieved. HsCRP and triglycerides achieved residual normality after log-transformation. Coefficients for log-transformed outcomes were exponentiated and reflect a percentage change in geometric mean of the outcome for each unit increase of the PTG factor score. Residual outliers were defined as Cook’s D > 4/*n*, where *n* is the sample size. Presence of residual outliers ranged from 0.8% for HbA1_C_ (*n* = 8) to 0.6.4% for pulse wave velocity (*n* = 61). During the diagnostic stage, non-linear relationships were investigated via inspection of the augmented component plus residual plot. Factors with plots suggestive of non-linear associations were transformed into a restricted cubic spline with three knots. A univariable linear regression model and subsequent likelihood ratio test (*p* < 0.05) was conducted between the PTG factor and the restricted cubic spline function of the factor with the cardiovascular health outcome to confirm which function better fit the data. Spearman’s correlation coefficients were generated between all variables of interest. Moderate associations were defined as Spearman’s correlation coefficients between 0.4 and 0.7 and strong associations were defined as > 0.7.

To address the aims of the study, variable selection procedures were conducted in line with recommendations from the literature (Heinze et al., 2018; Sauerbrei et al., 2020). First a screening step was conducted using BIF to assess model stability including all PTG factors and confounders. 1000 replications were used. To understand whether factors of PTG might be competing for selection through co-dependence, factors with BIF ≥ 30% were assessed for independence based on a chi^2^ analysis of BIF. Factors which were found to have been included due to co-dependence were removed in line with suggested practice for removal of co-dependent variables with weak associations (De Bin and Sauerbrei, 2018). Variables selected after screening were then subjected to weighted absolute least squares model averaging, bootstrapped with 1000 replications. Finally, to assess whether a model with individual or multiple PTG factors best fit the data, a likelihood ratio test was conducted (*p* < 0.05). Variable selection procedures were repeated excluding residual outliers.

Linear regression models were generated to confirm associations suggested by the variable selection procedure. PTG factors with identified non-linear associations were entered into models as restricted cubic spline functions. Models were estimated at a univariable level, at a multivariable level including confounders and at a multivariable level including confounders and excluding participants with probable anxiety, depression or PTSD. The third model is included due to potential confounding from the known curvilinear relationship between PTSD and PTG (Shakespeare-Finch and Lurie-Beck, 2014), and to assess whether the associations with PTG were apparent in the absence of mental ill health. All models were bootstrapped with at least 1000 replications and bias-corrected confidence intervals are reported.

A breakdown of the characteristics of participants who completed/did not complete the DPTGI can be found in Supplemental materials 2. 73.0% of participants (*n* = 756) completed the DPTGI on the day of their appointment or <12 months after their appointment, and 27.0% (*n* = 277) completed the DPTGI >12 months after their appointment. We have added a sensitivity analysis and produced four models: (a) participants who completed the PTGI on the day, (b) participants who completed it within 6 months, (c) participants who completed it within a year and (d) all participants who completed the measure. One model was only associated with the outcome when all participants (model d) were included in the analysis (appreciation of life-diastolic blood pressure) and one model was only significant when investigating participants who completed the assessment on the day (model a) (spiritual change-insulin resistance). Due to the moderate consistency of models across time, model d is presented in the results section of this paper.

Missing values ranged from three (PHQ; <1%) to 73 (insulin resistance; 6.3%). One item of the DPTGI was missing for 400 participants due to an administration error. Overall for the DPTGI, one item was missing for 430 participants, two items were missing for three participants and three items were missing for one participant. Values were imputed using two way imputation for participants with ≤3 items missing from the DPTGI (Van Ginkel et al., 2007). All other dependent variable missing data was handled using casewise deletion (range min: visceral adipose tissue *n* = 9 (<1.0%) max: LDL *n* = 50 (<5.0%)). Exclusion criteria for the current study included experiencing significant injury not related to his military service (*n* = 1), not completing the DPTGI (*n* = 108), >3 items missing on DPTGI (*n* = 2), or elevated HsCRP levels suggestive of current infection (HsCRP > 10) (*n* = 30).

## Results

1006/1145 participants of the ADVANCE cohort were included as part of this analysis. Table 1 shows the demographic characteristics of this sample. The median age of the sample was 34, with the majority of the sample being junior non-commissioned officers (*n* = 650; 64.6%), had not sustained a combat injury (*n* = 505; 50.2%), and were not on a cardiovascular or mental health medication of interest (*n* = 901; 90.6%). Sertraline was the most common mental health medication of interest (*n* = 24) and allopurinol was the most common cardiovascular medication of interest (*n* = 5). 24.5% (*n* = 246) reported probable depression, anxiety or PTSD. Spearman’s correlation coefficients between all PTG factors, confounders and cardiovascular health outcomes can be found in Supplemental materials 3. Moderate-strong correlations were noted between all PTG factors with the exception of SC, which had low-moderate correlation with other PTG factors.

**Table 1.** Demographic characteristics of sample.

	Overall sample
Total sample *No. (%)*	1006 (100.0%)
Demographics	
Age in years at assessment Median (IQR)	34 (30, 37)
Ethnicity	
White *No. (%)*	912 (90.4)
All other ethnic minorities *No. (%)*	94 (9.6)
Asian *No. (%)*	40 (3.8)
Black *No. (%)*	35 (3.8)
Mixed *No. (%)*	19 (2.0)
Rank	
Junior Non-Commissioned Officer rank/Other rank *No. (%)*	650 (70.8)
Senior Non-Commissioned Officer rank *No. (%)*	228 (21.3)
Commissioned Officer rank *No. (%)*	128 (7.9)
Combat injury	
Yes *No. (%)*	501 (46.5)
On Medication of interest	
Yes *No. (%)*	95 (9.5)
Cardiovascular medication *No. (%)*	25 (2.4)
Mental health medication *No. (%)*	70 (7.6)
Mental health	
Anxiety	
Yes (GAD7 score ≥ 10) *No. (%)*	165 (18.0)
Depression	
Yes (PHQ9 score ≥ 10) *No. (%)*	191 (20.6)
Post-Traumatic Stress Disorder	
Yes (PCL score ≥ 50) *No. (%)*	127 (14.1)
Deployment-related Post-Traumatic Growth (DPTGI)	
DPTGI Total score Median (IQR)	27 (16, 39)
DPTGI Appreciation of life (score range 0–9) Median (IQR)	5 (3, 7)
DPTGI Relating to others (score range 0–21) Median (IQR)	8 (4, 12)
DPTGI New possibilities (score range 0–15) Median (IQR)	7 (4, 10)
DPTGI Personal strength (score range 0–12) Median (IQR)	7 (4, 9)
DPTGI Spiritual change (score range 0–6) Median (IQR)	0 (0, 2)
Time in months between ADVANCE assessment and completing DPTGI Median (IQR)	0 (0, 12.7)
Haemodynamic functioning	
Heart rate (BPM) Median (IQR) *Normal resting heart rate 50–80BPM*	57.0 (51.7, 62.7)
Pulse wave velocity m/s Median (IQR) *Normal pulse wave velocity range: 4.2–9.4* *m/s*	7.8 (7.1, 8.8)
Inflammation	
Median HsCRP mmol/l Median (IQR) *Normal range* <*1.0 mmol/l*	0.9 (0.5, 1.8)
Cardiometabolic effects	
Diastolic blood pressure mmHg Median (IQR) *Normal diastolic blood pressure*: <*80 mmHg*	73.0 (67.3, 79.0)
Systolic blood pressure mmHg Median (IQR) *Normal systolic blood pressure*: <*120 mmHg*	129.0 (123.0, 137.0)
Total Cholesterol mg/dl Median (IQR) *Normal cholesterol*: <*200 mg/dl*	189.5 (166.3, 216.6)
Triglycerides mg/dl Median (IQR) *Normal triglycerides*: <*150 mg/dl*	97.4 (70.9, 141.7)
Triglycerides mg/dl Geometric mean (95% CI)	104.4 (100.0, 108.0)
High Density Lipoproteins mg/dl Median (IQR) *Normal High Density Lipoproteins: 40–60 mg/dl*	50.3 (42.5, 58.0)
Low Density Lipoproteins mg/dl Median (IQR) *Normal Low Density Lipoproteins*: <*130 mg/dl*	116.0 (96.7, 139.2)
HbA1C mg/dl Median (IQR) *Normal*: <*42 mmol/mol*	34.0 (32.0, 36.0)
eGDR (Insulin resistance) Median (IQR) *Indicative of metabolic syndrome ≤8.77 mg/kg/min*	9.8 (9.0, 10.4)
Visceral Adipose Tissue cm^2^ Median (IQR) *Normal*: <*100 cm* ^2^	86.3 (68.0, 114.1)

CI: confidence interval; DPTGI: Deployment-related Post-Traumatic Growth Inventory; eGDR: estimated glucose disposal rate; GAD: generalised anxiety disorder; IQR: interquartile range; PCL: post-traumatic stress disorder checklist; PHQ: Patient Health Questionnaire.

Variable selection procedures can be found in Supplemental materials 4. No associations were observed between any factor of PTG and HsCRP, systolic blood pressure, blood glucose, pulse wave velocity, resting heart rate or visceral adipose tissue. AOL was selected for outcomes of diastolic blood pressure, LDL, HDL and triglycerides. NP was selected for HDL only. PS was selected for DBP only. RTO was selected for total cholesterol only. SC was selected for outcomes of total cholesterol, triglycerides and insulin resistance. A non-linear relationship was identified between AOL and LDL. Co-dependency was noted between PS and SC with total cholesterol and AOL and NP with LDL. SC was retained for total cholesterol and AOL was retained for LDL.

**Figure 1.**
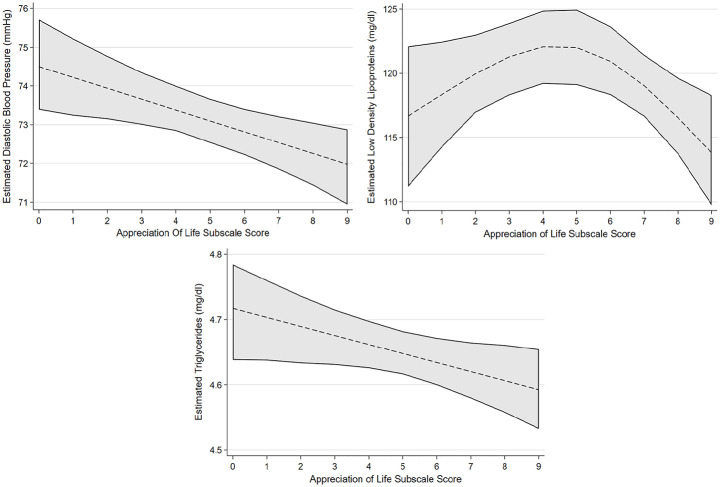
Estimated marginal effects and confidence intervals of the PTG factor Appreciation of Life associated with cardiovascular risk outcomes, confirmed in linear regression model.

**Figure 2.**
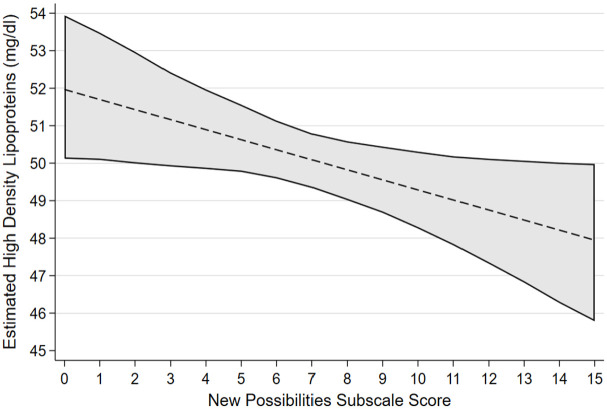
Estimated marginal effects and confidence intervals of the PTG factor New Possibilities associated with High Density Lipoproteins, confirmed in linear regression model.

**Figure 3.**
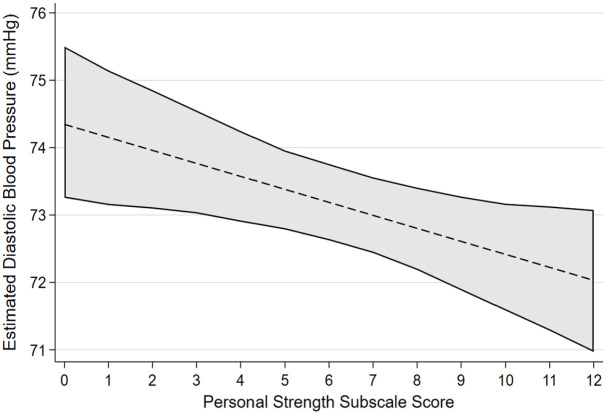
Estimated marginal effects and confidence intervals of the PTG factor Personal Strength associated with Diastolic Blood Pressure, confirmed in linear regression model.

**Figure 4.**
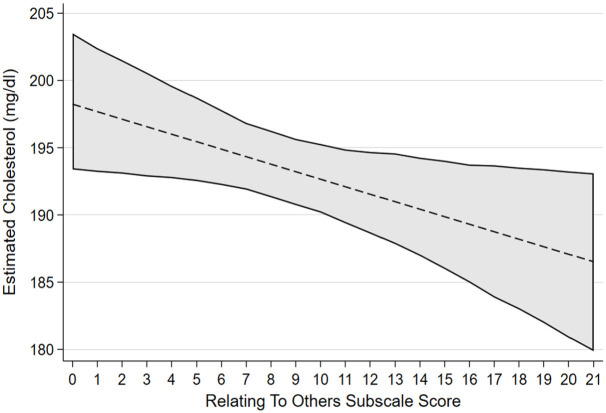
Estimated marginal effects and confidence intervals of the PTG factor Relating to Others associated with total cholesterol levels, confirmed in linear regression model.

Table 2 reports the associated regression coefficients and 95% bias-corrected confidence intervals between each PTG factor and cardiovascular health factors selected by variable selection procedures with linear associations. After accounting for confounders, greater scores on the factor of AOL were confirmed as having an association with lower diastolic blood pressure, lower triglyceride levels and having a non-linear association with LDL. Greater scores on the factor of NP were confirmed to be associated with lower HDL. Greater scores on the factor of PS were confirmed to have an association with lower diastolic blood pressure. Greater scores on the factor of RTO were confirmed to have an association with lower total cholesterol. Figures 1 to 4 presents the marginal effect associations between PTG factors and cardiovascular health indicators confirmed to have an association via linear regression modelling. Limited evidence for associations were present for some factors due to being selected by variable selection procedures but not confirmed by linear regression modelling. Supplemental materials 5 shows the marginal effects that were not confirmed during linear regression modelling.

**Table 2.** Robust regression coefficients for Post-traumatic Growth factors on cardiovascular risk outcomes with linear relationships.

	Post-traumatic growth factor	Model 1* coefficient (95% bias-corrected CI)	Model 2** coefficient (95% bias-corrected CI)	Model 3*** coefficient (95% bias-corrected CI)
Diastolic blood pressure	Appreciation of life (score range 0–9)	−0.262 (−0.480, −0.070)	−0.281 (−0.495, −0.099)	−0.349 (−0.585, −0.121)
	Personal strength (score range 0–6)	−0.226 (−0.402, −0.075)	−0.193 (−0.364, −0.041)	−0.202 (−0.390, −0.021)
High Density Lipoproteins	Appreciation of life (score range 0–9)	0.540 (0.159, 0.942)	0.359 (−0.053, 0.756)	0.351 (−0.096, 0.758)
	New possibilities (score range 0–15)	−0.388 (−0.650, −0.156)	−0.268 (−0.534, −0.032)	−0.359 (−0.647, −0.055)
Estimated Glucose Disposal Rate (Insulin Resistance)	Relating to others (score range 0–21)	0.002 (−0.014, 0.017)	0.005 (−0.010, 0.019)	0.004 (−0.013, 0.022)
	Spiritual change (score range 0–6)	−0.048 (−0.102, 0.003)	−0.029 (−0.081, 0.191)	−0.023 (−0.080, 0.030)
Total cholesterol	Relating to others^†^ (score range 0–21)	−0.558 (−1.092, −0.069)	−0.558 (−1.082, −0.057)	−0.687 (−1.186, −0.103)
	Spiritual change^†^ (score range 0–6)	2.337 (0.440, 4.476)	1.706 (−0.166, 3.847)	2.252 (0.066, 4.445)
Triglycerides^††^	Appreciation of life (score range 0–9)	−1.519% (−2.763, 0.666)	−1.376% (−2.615, −0.039)	−1.177% (−2.649, 0.198)
	Spiritual change (score range 0–6)	1.179% (−0.991, 3.784)	0.488% (−1.629, 2.786)	0.047% (−1.470, 3.615)

* Univariable model including PTG subscale score only.

** Model including PTG subscale score and confounders (age at assessment, combat injury, medication use and socioeconomic status).

*** Model including PTG subscale score and confounders (age at assessment, combat injury, medication use, socioeconomic status). Excludes those meeting threshold scores for probable anxiety, depression or PTSD).

† Both PTG subscale scores are included in a single model.

†† Regression model was conducted on log-transformed triglycerides and exponentiated coefficients, reflecting percentage change in geometric mean, which are reported here.

In the model excluding those with mental illness (e.g. probable depression, anxiety or PTSD), all associations identified in model 2 were retained with the exception of the association between triglycerides and AOL. Greater scores on the factor of SC were confirmed to have an association with total cholesterol in the model excluding those with mental illness only.

## Discussion

In this study we hypothesised that factors of PTG would be associated with a better cardiovascular health profile in the ADVANCE study cohort. We found that factors of PTG were associated with cardiometabolic effects and haemodynamic functioning but were not associated with inflammation (specifically inflammatory marker HsCRP). Our hypothesis was supported by our observations that the majority of factors of PTG were associated with better cardiovascular health indicators. Linear regression models confirmed an association between greater scores on the AOL factor with decreased diastolic blood pressure, decreased triglyceride levels and had a non-linear association with LDL cholesterol. Greater scores on the PS factor were associated with decreased diastolic blood pressure. Greater scores on the RTO factor were associated with lower total cholesterol levels. However, our hypothesis was also partially rejected as there were also associations between PTG and worse cardiovascular health indicators. Greater scores on the NP factor were associated with decreased HDL cholesterol. Greater scores on the SC factor were associated with increased total cholesterol, though only when those with probable mental illness were excluded. The strength of all associations between PTG factors and cardiovascular health indicators were small to modest.

### Post-traumatic growth, mental illness and the cardiovascular system

It is of note that almost all associations between PTG and cardiovascular health outcomes were present even in the absence of participants with probable mental illness as observed in model three of our analysis which excluded those with probable depression, anxiety, and PTSD. This suggests that these positive psychological outcomes are associated with cardiovascular health even amongst those with relatively good psychological well-being, supporting evidence that measurement of PTG is not simply measuring the absence of mental illness. The utility of interventions that are shown to increase positive psychological constructs (Dubois et al., 2012; Mohammadi et al., 2020; Murphy et al., 2017; Nikrahan et al., 2016) may theoretically expand to positive effects on cardiovascular health, even amongst those with relatively good mental health, though longitudinal research would be needed to confirm this.

### Spirituality and the cardiovascular system

Spirituality and religiousness are generally perceived to be protective factors in CVD. However, a meta-analysis investigating the physiological markers associated with religiosity/spirituality also reported that diabetic risk markers including diabetes status, insulin resistance and fasting blood glucose were increased in those who reported being religious/spiritual (Shattuck and Muehlenbein, 2020). In our study, SC was associated with worse cardiometabolic health indicators, specifically greater levels of total cholesterol in the model excluding participants with probable mental illnesses and limited evidence was also noted for associations with greater insulin resistance. It could be that those who experienced SC were more likely to come from demographics at higher risk of CVD, such as lower socioeconomic status (Schieman, 2010) or come from ethnic minority groups (Agbonlahor et al., 2023), though further investigation is required to understand the mechanisms behind this relationship.

### New possibilities and lifestyle factors

The NP factor was associated with lower levels of HDL cholesterol, which is generally considered as ‘good’ cholesterol. This factor includes items such as developing new interests, being able to make changes when necessary, seeing new opportunities, establishing new paths, and perceiving those paths/options to be better than prior to the trauma. It is plausible that establishing new hobbies or interests could be correlated with increased opportunities to drink alcohol or eating at social events, though no research appears to exist on this link. On the other hand, it is also plausible that persons experiencing a large amount of growth on the NP factor might engage in more physically active hobbies, such as sports, or home cooking/positive changes to diet. Experiencing greater growth on the NP subscale might be associated with both a mixture of poorer and better lifestyle factors. Future research would benefit from establishing whether there are differences in lifestyle factors such as diet, physical activity, and alcohol use/smoking/drug use between the factors of PTG.

### Strengths/limitations

Strengths of the study include investigation of a range of cardiovascular health indicators, a reasonably large sample size and use of best-practice statistical methodology for variable selection (Heinze et al., 2018; Sauerbrei et al., 2020). However, this study also has a number of limitations. These results are based on cross-sectional analyses, therefore causation cannot be inferred. Whilst the majority of the ADVANCE cohort completed the DPTGI on the day of their assessment, a portion of the sample reported PTG over a year after their appointment. This is a limitation of the study, though sensitivity analyses suggested that this did not have a major impact on associations for most findings. Missing data required the use of two-way imputation at an item level for the DPTGI. PTG was defined as specifically growth relating to any military deployments to Iraq/Afghanistan, which would not acknowledge PTG relating to any other traumatic event, which could confound associations in this study. It is likely that factors such as alcohol use, smoking and physical exercise mediate the associations observed in our study. Mediation analysis was beyond the scope of this current paper. The sample was limited to only male (sex) personnel, and findings may not translate to the female experience of PTG (Vishnevsky et al., 2010). Finally, whilst the five factors of PTG have been validated amongst a range of demographics including military/veterans (Lee et al., 2010), questions exist regarding the distinctness of the factors from one another (Silverstein et al., 2018), which might explain why co-dependence was noted between some factors in our variable selection procedures. It is possible that a more parsimonious understanding of PTG might be more beneficial to understanding the impact of PTG on physical health.

## Conclusions

PTG is associated with mostly beneficial cardiometabolic effects and haemodynamic functioning in a sample of male UK military personnel who deployed to Afghanistan, however associations with deleterious cardiovascular health indicators are also noted. PTG is not associated with inflammation (specifically marker HsCRP). Further investigation is required to understand why aspects of PTG are associated with both positive and negative cardiovascular health indicators, and to understand whether the associations observed translate to changes in a person’s cardiovascular disease risk profile in the long-term. Investigation into interventions that elicit aspects of positive psychological thriving and assess their impact on long-term cardiovascular health is encouraged.

## Supplemental Material

sj-docx-1-hpq-10.1177_13591053241240196 – Supplemental material for The underlying mechanisms by which Post-Traumatic Growth is associated with cardiovascular health in male UK military personnel: The ADVANCE cohort study Supplemental material, sj-docx-1-hpq-10.1177_13591053241240196 for The underlying mechanisms by which Post-Traumatic Growth is associated with cardiovascular health in male UK military personnel: The ADVANCE cohort study by Daniel Dyball, Alexander N Bennett, Susie Schofield, Paul Cullinan, Christopher J Boos, Anthony MJ Bull, Sharon AM Stevelink and Nicola T Fear in Journal of Health Psychology
