# Supplementary material for: The underlying mechanisms by which Post-Traumatic Growth is associated with cardiovascular health in male UK military personnel: The ADVANCE cohort study
Source: J Health Psychol. 2024 Apr 11;30(6):1160–76. doi: 10.1177/13591053241240196 (PMC12053277; doi:10.1177/13591053241240196)

Supplementary materials 1: Classification of normal ranges for cardiovascular outcomes

| **Outcome** | **Range** | **Reference** |
| --- | --- | --- |
| Cholesterol (total) | Desirable: <200mg/dl  Borderline high: 200-239mg/dl  High: ≥240mg/dl | National Institutes of Health. (2001). ATP III guidelines at-a-glance quick desk reference. *NIH publication*, 01-3305. |
| Diastolic blood pressure | Normal: <80mmHg  Prehypertensive: 80-89 mmHg  Stage 1 hypertension 90-99 mmHg  Stage 2 hypertension ≥100 mmHg | Chobanian, A. V., Bakris, G. L., Black, H. R., Cushman, W. C., Green, L. A., Izzo Jr, J. L., ... & National High Blood Pressure Education Program Coordinating Committee. (2003). The seventh report of the joint national committee on prevention, detection, evaluation, and treatment of high blood pressure: the JNC 7 report. *Jama*, *289*(19), 2560-2571. |
| Estimated Glucose Disposal Rate (Insulin Resistance) | Indicative of metabolic syndrome: ≤8.77mg/kg/min | Chillaron, J. J., Goday, A., Flores-Le-Roux, J. A., Benaiges, D., Carrera, M. J., Puig, J., ... & Pedro-Botet, J. (2009). Estimated glucose disposal rate in assessment of the metabolic syndrome and microvascular complications in patients with type 1 diabetes. *The journal of clinical endocrinology & metabolism*, *94*(9), 3530-3534. |
| HbA1_C_ | Normal: <42mmol/mol | The International Expert Committee. International Expert Committee  report on the role of the A1c assay in the diagnosis of diabetes.  *Diabetes Care* 2009;32:1327–34 |
| HDL | Low (risk factor): <40mg/dl  High (negative risk factor):>60mg/dl | National Institutes of Health. (2001). ATP III guidelines at-a-glance quick desk reference. *NIH publication*, 01-3305. |
| High Sensitivity C-Reactive Protein | Low risk: <1mg/l  Average: 1-3mg/l  High risk: >3mg/l | Pearson, T. A., Mensah, G. A., Alexander, R. W., Anderson, J. L., Cannon III, R. O., Criqui, M., ... & Vinicor, F. (2003). Markers of inflammation and cardiovascular disease: application to clinical and public health practice: a statement for healthcare professionals from the Centers for Disease Control and Prevention and the American Heart Association. *circulation*, *107*(3), 499-511. |
| LDL | Optimal: <100 mg/dl  Near optimal: 100-129mg/dl  Borderline high: 130-159mg/dl  High 160-189mg/dl  Very high: ≥190mg/dl | National Institutes of Health. (2001). ATP III guidelines at-a-glance quick desk reference. *NIH publication*, 01-3305. |
| Pulse wave velocity | Optimal range: 6.6m/s (+2 SD 4.4–8.9)  Normal range: 6.8m/s (+2 SD 4.2–9.4)  High normal range: 7.1m/s (+2 SD 4.5–9.7)  Grade 1 Hypertension range: 7.3m/s (+2 SD 4.0–10.7)  Grade 2 Hypertension range: 8.2m/s (+2 SD 3.3–13.0) | Reference Values for Arterial Stiffness' Collaboration. (2010). Determinants of pulse wave velocity in healthy people and in the presence of cardiovascular risk factors:‘establishing normal and reference values’. *European heart journal*, *31*(19), 2338-2350. |
| Resting heart rate | Normal: 50-80bpm | Quer, G., Gouda, P., Galarnyk, M., Topol, E. J., & Steinhubl, S. R. (2020). Inter-and intraindividual variability in daily resting heart rate and its associations with age, sex, sleep, BMI, and time of year: Retrospective, longitudinal cohort study of 92,457 adults. *Plos one*, *15*(2), e0227709. |
| Systolic blood pressure | Normal: <120 mmHg  Prehypertensive: 120-139 mmHg  Stage 1 Hypertension: 140-159 mmHg  Stage 2 Hypertension: ≥160 mmHg | Chobanian, A. V., Bakris, G. L., Black, H. R., Cushman, W. C., Green, L. A., Izzo Jr, J. L., ... & National High Blood Pressure Education Program Coordinating Committee. (2003). The seventh report of the joint national committee on prevention, detection, evaluation, and treatment of high blood pressure: the JNC 7 report. *Jama*, *289*(19), 2560-2571. |
| Triglycerides | Normal: <150mg/dl  Borderline high: 150-199mg/dl  High: 200-499mg/dl  Very high: ≥500mg/dl | National Institutes of Health. (2001). ATP III guidelines at-a-glance quick desk reference. *NIH publication*, 01-3305. |
| Visceral Adipose Tissue | Normal: <100cm^2^  Increased risk: 100-160cm^2^  High risk: ≥160cm^2^ | Kelly, T. L. (2010). Practical and technical advantages of DXA visceral fat assessment compared with computed tomography. *Age*, *36*(42.3), 50. |

Supplementary materials 2: Breakdown of ADVANCE study participants who did/did not complete the deployment related post traumatic growth inventory

Completed ADVANCE assessment

*n*=1145

Completed DPTGI on ADVANCE assessment day

*n*=565

Completed DPTGI after ADVANCE assessment day

*n*=472

Did not complete DPTGI

*n*=108

Age median (IQR): 31.5 (28, 36)

Combat injury *n (%)*: 57 (52.8)

All other ethnic minorities *n (%): 12 (11.1)*

Low rank *n (%)*: 82 (75.9)

Mid rank *n (%)*: (17 (15.8)

Officer rank *n (%)*: 9 (8.3)

Medication of interest *n (%)*: 14 (13.0)

Depression *n (%)*: 20 (18.7)

Anxiety *n (%)*: 14 (13.1)

PTSD *n (%)*: 11 (10.2)

Age median (IQR): 32 (29, 36)

Combat injury *n (%)*: 271 (46.7)

All other ethnic minorities *n (%): 50 (8.6)*

Low rank *n (%)*: 373 (64.3)

Mid rank *n (%)*: 128 (22.1)

Officer rank *n (%)*: 79 (13.6)

Medication of interest *n (%)*: 56 (9.7)

Depression *n (%)*: 84 (14.5)

Anxiety *n (%)*: 68 (11.7)

PTSD *n (%)*: 51 (8.8)

Age median (IQR): 34 (31, 38)

Combat injury *n (%)*: 308 (54.5)

All other ethnic minorities *n (%): 58 (10.3)*

Low rank *n (%)*: 381 (67.4)

Mid rank *n (%)*: 125 (22.1)

Officer rank *n (%)*: 59 (10.5)

Medication of interest *n (%)*: 56 (9.9)

Depression *n (%)*: 133 (23.6)

Anxiety *n (%)*: 114 (20.2)

PTSD *n (%)*: 91 (16.2)

Supplementary materials 3: Regression coefficients for Post-traumatic Growth factors on cardiovascular risk outcomes with linear relationships

|  | **Post-traumatic growth factor** | **Only participants who completed all measures on assessment day (n=551)** | **Only participants who completed all measures within 180 days of assessment day (n=598)** | **Only participants who completed all measures within 365 days of assessment day (n=739)** | **All participants (n=1006)** |
| --- | --- | --- | --- | --- | --- |
| **Diastolic blood pressure** | Appreciation of lifeⴕ (score range 0-9) | -0.142 (-0.383, 0.099) | -0.146 (-0.392, 0.081) | -0.150 (-0.351, 0.056) | **-0.281 (-0.495, -0.099)** |
|  | Personal strengthⴕ (score range 0-6) | **-0.214 (-0.394, -0.-12)** | **-0.195 (-0.384, -0.006)** | **-0.162 (-0.329, 0.014)** | **-0.193 (-0.364, -0.041)** |
|  |  |  |  |  |  |
| **High Density Lipoproteins** | Appreciation of life (score range 0-9) | 0.291 (-0.129, 0.693) | 0.193 (-0.192, 0.602) | 0.312 (-0.056, 0.675) | 0.359 (-0.053, 0.756) |
|  | New possibilities (score range 0-15) | -0.155 (-0.420, 0.120) | **-0.173 (-0.436, -0.075)** | -0.242 (-0.497, 0.012) | **-0.268 (-0.534, -0.032)** |
| **Estimated Glucose Disposal Rate (Insulin Resistance)** | Relating to others (score range 0-21) | 0.011 (-0.001, 0.023) | 0.009 (-0.003, 0.022) | 0.010 (-0.003, 0.020) | 0.005 (-0.010, 0.019) |
|  | Spiritual change (score range 0-6) | **0.010 (-0.033, -0.055)** | 0.004 (-0.040, 0.043) | -0.007 (-0.046, 0.029) | -0.029 (-0.081, 0.191) |
|  |  |  |  |  |  |
| **Total cholesterol** | Relating to othersⴕ (score range 0-21) | **-0.563 (-1.109, -0.042)** | **-0.604 (-1.118, -0.077)** | **-0.525 (-0.952, -0.044)** | **-0.558 (-1.082, -0.057)** |
|  | Spiritual changeⴕ (score range 0-6) | 1.092 (-0.746, 2.916) | 1.155 (-0.509, 2.791) | 1.418 (-0.170, 2.943) | 1.706 (-0.166, 3.847) |
| **Triglycerides**ⴕⴕ | Appreciation of life (score range 0-9) | **-2.092 (-3.530, -0.721)** | **-1.907% (-3.241, -0.597)** | **-1.551% (-2.670, -0.316)** | **-1.376% (-2.615, -0.039)** |
|  | Spiritual change | -1.351 (-3.327, 0.828) | -1.474% (-3.340, 0.780) | -0.796% (-2.639, 0.985) | 0.488% (-1.629, 2.786) |

Supplementary materials 4: Spearman correlation matrix between Post-Traumatic Growth scores, metabolic effects, inflammation, haemodynamic functioning and confounders

|  | PTG: DPTGI-Appreciation of life | PTG: DPTGI-New possibilities | PTG: DPTGI-Personal Strength | PTG: DPTGI-Relating to others | PTG: DPTGI-Spiritual Change | Anxiety-GAD case | Depression-PHQ case | PTSD-PCL case | Age at assessment | Combat injury | Black and other minority ethnicities | Medication | Socioeconomic status | Diastolic blood pressure | Heart rate | Systolic blood pressure | Pulse wave velocity | HsCRP | Cholesterol | HbA1C | HDL | Insulin resistance | LDL | Triglycerides | Visceral Fat |
| --- | --- | --- | --- | --- | --- | --- | --- | --- | --- | --- | --- | --- | --- | --- | --- | --- | --- | --- | --- | --- | --- | --- | --- | --- | --- |
| PTG: DPTGI-Appreciation of life | 1.00 | - | - | - | - | - | - | - | - | - |  | - | - | - | - | - | - | - | - | - | - | - | - | - | - |
| PTG: DPTGI-New possibilities | 0.67 | 1.00 | - | - | - | - | - | - | - | - |  | - | - | - | - | - | - | - | - | - | - | - | - | - | - |
| PTG: DPTGI-Personal Strength | 0.67 | 0.72 | 1.00 | - | - | - | - | - | - | - |  | - | - | - | - | - | - | - | - | - | - | - | - | - | - |
| PTG: DPTGI-Relating to others | 0.69 | 0.70 | 0.66 | 1.00 | - | - | - | - | - | - |  | - | - | - | - | - | - | - | - | - | - | - | - | - | - |
| PTG: DPTGI-Spiritual Change | 0.40 | 0.42 | 0.39 | 0.48 | 1.00 | - | - | - | - | - |  | - | - | - | - | - | - | - | - | - | - | - | - | - | - |
| Anxiety-GAD case | -0.16 | -0.10 | -0.12 | -0.10 | -0.03 | 1.00 | - | - | - | - |  | - | - | - | - | - | - | - | - | - | - | - | - | - | - |
| Depression-PHQ case | -0.24 | -0.16 | -0.17 | -0.18 | -0.08 | 0.60 | 1.00 | - | - | - |  | - | - | - | - | - | - | - | - | - | - | - | - | - | - |
| PTSD-PCL case | -0.15 | -0.06 | -0.07 | -0.09 | -0.03 | 0.63 | 0.62 | 1.00 | - | - |  | - | - | - | - | - | - | - | - | - | - | - | - | - | - |
| Age at assessment | 0.00 | -0.10 | -0.10 | 0.00 | 0.03 | -0.02 | 0.00 | -0.02 | 1.00 | - |  | - | - | - | - | - | - | - | - | - | - | - | - | - | - |
| Combat injury | 0.05 | 0.21 | 0.06 | 0.08 | 0.09 | 0.11 | 0.11 | 0.08 | -0.06 | 1.00 |  | - | - | - | - | - | - | - | - | - | - | - | - | - | - |
| Black and other minority ethnicities | 0.07 | 0.14 | 0.10 | 0.17 | 0.27 | -0.09 | -0.11 | -0.06 | 0.04 | -0.01 | 1.00 |  |  |  |  |  |  |  |  |  |  |  |  |  | -0.02 |
| Medication | -0.09 | -0.04 | -0.09 | -0.01 | 0.02 | 0.31 | 0.27 | 0.28 | 0.06 | 0.07 | -0.02 | 1.00 | - | - | - | - | - | - | - | - | - | - | - | - | - |
| Socioeconomic status | 0.05 | -0.15 | -0.10 | -0.03 | -0.04 | -0.16 | -0.14 | -0.16 | 0.47 | -0.12 | -0.15 | -0.06 | 1.00 | - | - | - | - | - | - | - | - | - | - | - | - |
| Diastolic blood pressure | -0.10 | -0.08 | -0.11 | -0.03 | -0.01 | 0.01 | 0.01 | -0.02 | 0.20 | -0.05 | 0.11 | 0.08 | 0.10 | 1.00 | - | - | - | - | - | - | - | - | - | - | - |
| Heart rate | -0.06 | 0.00 | -0.02 | -0.05 | -0.02 | 0.14 | 0.14 | 0.12 | 0.00 | 0.15 | 0.00 | 0.12 | -0.14 | 0.22 | 1.00 | - | - | - | - | - | - | - | - | - | - |
| Systolic blood pressure | -0.04 | -0.06 | -0.04 | -0.01 | -0.02 | 0.08 | 0.08 | 0.01 | 0.21 | -0.01 | 0.13 | 0.10 | -0.01 | 0.54 | 0.14 | 1.00 | - | - | - | - | - | - | - | - | - |
| Pulse wave velocity | -0.02 | -0.07 | -0.03 | -0.04 | -0.06 | 0.04 | 0.08 | 0.04 | 0.11 | 0.01 | -0.01 | 0.05 | 0.02 | 0.08 | 0.03 | 0.16 | 1.00 | - | - | - | - | - | - | - | - |
| HsCRP | 0.01 | 0.05 | 0.01 | 0.02 | 0.00 | 0.05 | 0.08 | 0.02 | 0.04 | 0.08 | 0.03 | 0.09 | -0.07 | 0.17 | 0.18 | 0.16 | 0.01 | 1.00 | - | - | - | - | - | - | - |
| Cholesterol | -0.05 | -0.10 | -0.12 | -0.07 | 0.00 | -0.04 | 0.03 | -0.06 | 0.25 | -0.06 | 0.03 | -0.02 | 0.17 | 0.21 | 0.10 | 0.13 | -0.01 | 0.04 | 1.00 | - | - | - | - | - | - |
| HbA1C | -0.02 | -0.03 | -0.01 | 0.03 | 0.06 | -0.03 | -0.03 | 0.00 | 0.18 | -0.09 | 0.20 | 0.04 | 0.05 | 0.13 | -0.03 | 0.05 | -0.02 | 0.07 | 0.19 | 1.00 | - | - | - | - | - |
| HDL | 0.04 | -0.07 | -0.01 | -0.01 | 0.03 | -0.14 | -0.12 | -0.12 | 0.13 | -0.07 | 0.04 | -0.06 | 0.14 | -0.09 | -0.18 | 0.01 | 0.05 | -0.21 | 0.18 | 0.00 | 1.00 | - | - | - | - |
| Insulin resistance | 0.06 | 0.06 | 0.04 | 0.06 | -0.01 | -0.08 | -0.16 | -0.11 | -0.25 | -0.08 | 0.02 | -0.19 | -0.05 | -0.31 | -0.22 | -0.32 | -0.10 | -0.36 | -0.18 | -0.20 | 0.25 | 1.00 | - | - | - |
| LDL | -0.03 | -0.07 | -0.09 | -0.06 | 0.01 | -0.05 | 0.02 | -0.05 | 0.19 | -0.07 | 0.02 | -0.05 | 0.13 | 0.16 | 0.08 | 0.09 | -0.01 | 0.06 | 0.93 | 0.18 | -0.02 | -0.18 | 1.00 | - | - |
| Triglycerides | -0.12 | -0.04 | -0.07 | -0.03 | -0.04 | 0.12 | 0.14 | 0.13 | 0.14 | 0.08 | 0.00 | 0.15 | 0.03 | 0.27 | 0.25 | 0.17 | 0.01 | 0.17 | 0.39 | 0.12 | -0.34 | -0.40 | 0.26 | 1.00 | - |
| Visceral Fat | -0.04 | -0.05 | -0.05 | -0.03 | 0.01 | 0.10 | 0.16 | 0.07 | 0.33 | 0.10 | -0.02 | 0.16 | 0.02 | 0.27 | 0.25 | 0.30 | 0.07 | 0.36 | 0.19 | 0.05 | -0.26 | -0.73 | 0.17 | 0.44 | 1.00 |

Supplementary materials 5: Variable Selection Procedures

| **Outcome group** | **PTG factor** | **Outcome** | **Evidence of non-linear relationship?** | **Bootstrap Inclusion Frequencies** | **Bootstrap Inclusion Frequencies (excluding outliers)** | **Co-dependence?** | **WALS t-score (1-Std. error bands)** | **WALS t-score (1-Std. error bands) (excluding outliers)** | **Multiple symptom model likelihood ratio chi^2^ test (*p*-value)** | **Multiple symptom model likelihood ratio chi^2^ test (*p*-value) (excluding outliers)** |
| --- | --- | --- | --- | --- | --- | --- | --- | --- | --- | --- |
| **Inflammation** | Appreciation of life | HsCRP | No | 6.00 | 6.10 | - | - | - |  |  |
|  | New possibilities | HsCRP | No | 23.00 | 19.40 | - | - | - |  |  |
|  | Personal strength | HsCRP | No | 11.60 | 5.90 | - | - | - |  |  |
|  | Relating to others | HsCRP | No | 7.00 | 8.20 | - | - | - |  |  |
|  | Spiritual change | HsCRP | No | 5.90 | 9.30 | - | - | - |  |  |
| **Haemodynamic functioning** | **Appreciation of life** | **Diastolic blood pressure** | **No** | **48.20** | **20.80** | **No** | **-1.65 (-0.26, -0.06)** | **-0.77 (-0.15, 0.02)** | **0.30 (.58)** | **1.60 (0.21)** |
|  | New possibilities | Diastolic blood pressure | No | 15.50 | 16.10 | - | - | - |  |  |
|  | **Personal strength** | **Diastolic blood pressure** | **No** | **17.40** | **34.30** | **No** | **-0.73 (-0.14, 0.02)** | **-1.27 (-0.16, -0.01)** | **2.76 (.10)** | **0.42 (0.51)** |
|  | Relating to others | Diastolic blood pressure | No | 11.30 | 11.30 | - | - | - |  |  |
|  | Spiritual change | Diastolic blood pressure | No | 13.80 | 7.50 | - | - | - |  |  |
|  | Appreciation of life | Pulse wave velocity | No | 5.00 | 14.50 | - | - | - |  |  |
|  | New possibilities | Pulse wave velocity | No | 10.10 | 18.20 | - | - | - |  |  |
|  | Personal strength | Pulse wave velocity | No | 6.20 | 5.70 | - | - | - |  |  |
|  | Relating to others | Pulse wave velocity | No | 8.80 | 10.00 | - | - | - |  |  |
|  | Spiritual change | Pulse wave velocity | No | 25.50 | 19.50 | - | - | - |  |  |
|  | Appreciation of life | Resting heart rate | No | 10.20 | 14.80 | - | - | - |  |  |
|  | New possibilities | Resting heart rate | Yes | 7.60  Term2 2.70 | 24.30  Term2 7.60 | - | - | - |  |  |
|  | Personal strength | Resting heart rate | No | 11.40 | 10.60 | - | - | - |  |  |
|  | Relating to others | Resting heart rate | No | 24.60 | 16.70 | - | - | - |  |  |
|  | Spiritual change | Resting heart rate | No | 8.50 | 38.20 | - | -0.83 (-0.20, 0.02) | -0.54 (-0.19, 0.06) |  |  |
|  | Appreciation of life | Systolic blood pressure | No | 9.90 | 6.60 | - | - | - |  |  |
|  | New possibilities | Systolic blood pressure | No | 37.80 | 14.40 | - | -0.96 (-0.42, 0.01) | -0.82 (-0.43, 0.04) |  |  |
|  | Personal strength | Systolic blood pressure | No | 10.00 | 6.10 | **-** | **-** | **-** |  |  |
|  | Relating to others | Systolic blood pressure | No | 9.30 | 5.40 | - | - | - |  |  |
|  | Spiritual change | Systolic blood pressure | No | 8.70 | 12.10 | - | - | - |  |  |
| **Metabolic effects** | Appreciation of life | Cholesterol (total) | No | 10.30 | 12.50 | - | - | - |  |  |
|  | New possibilities | Cholesterol (total) | No | 12.90 | 19.10 | - | - | - |  |  |
|  | Personal strength | Cholesterol (total) | No | 23.70 | 31.90 | Yes | - | - |  |  |
|  | **Relating to others** | **Cholesterol (total)** | **No** | **25.50** | **30.00** | **No** | **-1.86 (-0.62, -0.19)** | **1.76 (0.45, 1.65)** | **3.91 (.05)** | **3.31 (.07)** |
|  | **Spiritual change** | **Cholesterol (total)** | **No** | **47.20** | **42.10** | **Yes** | **1.26 (0.45, 2.07)** | **-0.39 (-0.56, -0.22)** | **4.86 (.03)** | **6.41 (.01)** |
|  | Appreciation of life | HbA1c | No | 18.30 | 6.60 | - | - | - |  |  |
|  | New possibilities | HbA1c | No | 26.40 | 38.50 | - | 0.43 (-0.03, 0.08) | -0.96 (-0.03, 0.00) |  |  |
|  | Personal strength | HbA1c | No | 2.60 | 12.80 | - | - | - |  |  |
|  | Relating to others | HbA1c | No | 5.40 | 15.00 | - | - | - |  |  |
|  | Spiritual change | HbA1c | No | 7.90 | 10.00 | - |  |  |  |  |
|  | **Appreciation of life** | **High Density Lipoproteins** | **No** | **31.80** | **67.90** | **-** | **1.55 (0.09, 0.42)** | **2.44 (0.21, 0.49)** | **4.57 (.03)** | **11.78 (<.01)** |
|  | **New possibilities** | **High Density Lipoproteins** | **No** | **47.20** | **83.20** | **-** | **-1.75 (-0.29, -0.08)** | **-2.90 (-0.39, -0.19)** | **3.45 (.06)** | **6.51 (.01)** |
|  | Personal strength | High Density Lipoproteins | No | 8.30 | 7.20 | - | - | - |  |  |
|  | Relating to others | High Density Lipoproteins | No | 9.60 | 6.30 | - | - | - |  |  |
|  | Spiritual change | High Density Lipoproteins | No | 9.00 | 6.00 | - | - | - |  |  |
|  | Appreciation of life | Insulin resistance | No | 6.50 | 8.00 | - | - | - |  |  |
|  | New possibilities | Insulin resistance | No | 13.50 | 10.30 | - | - | - |  |  |
|  | Personal strength | Insulin resistance | No | 17.80 | 16.60 | - | - | - |  |  |
|  | **Relating to others** | **Insulin resistance** | No | **33.30** | **50.00** | - | **1.32 (0.00, 0.01)** | **1.81 (0.00, 0.01)** | **3.15 (.08)** | **1.06 (.30)** |
|  | **Spiritual change** | **Insulin resistance** | **No** | **35.50** | **10.90** | - | **-1.52 (-0.05, -0.01)** | **-0.94 (-0.03, 0.00)** | **2.28 (.13)** | **4.26 (.04)** |
|  | **Appreciation of life** | **Low Density Lipoproteins** | **Yes** | **61.70**  **Term2 28.20** | **35.70**  **Term2 11.20** | **Yes** | **Term1 1.90 (0.78, 2.53)**  **Term2 -2.12 (-3.14, -1.13)** | **Term 1 1.72 (0.54, 2.05)**  **Term 2 -1.60 (-2.34, -0.54)** | **-** | **-** |
|  | New possibilities | Low Density Lipoproteins | No | 11.50 | 12.40 | - |  |  |  |  |
|  | Personal strength | Low Density Lipoproteins | No | 34.60 | 38.70 | Yes |  |  |  |  |
|  | Relating to others | Low Density Lipoproteins | No | 21.80 | 31.90 | - | -0.47 (-0.28, 0.10) | -0.97 (-0.32, 0.00) |  |  |
|  | Spiritual change | Low Density Lipoproteins | No | 22.60 | 26.50 | - |  |  |  |  |
|  | **Appreciation of life** | **Trigylcerides** | No | **58.30** | **86.60** | - | **-2.11 (-0.02, -0.01)** | **-2.81 (-0.02, -0.01)** | **2.16 (.14)** | **0.92 (0.34)** |
|  | New possibilities | Trigylcerides | Yes | 22.60  Term2  7.20 | 19.40  Term2 4.90 | - |  |  |  |  |
|  | Personal strength | Trigylcerides | No | 6.00 | 9.70 | - |  |  |  |  |
|  | Relating to others | Trigylcerides | No | 15.30 | 17.10 | - |  |  |  |  |
|  | **Spiritual change** | **Trigylcerides** | **No** | **32.40** | **11.80** | **-** | **1.28 (0.00, 002)** | **0.86 (-0.00, 0.02)** | **6.92 (<.01)** | **11.83 (<.01)** |
|  | Appreciation of life | Visceral Adipose Tissue | No | 8.70 | 6.90 | - | - | - |  |  |
|  | New possibilities | Visceral Adipose Tissue | No | 24.20 | 13.90 | - |  |  |  |  |
|  | Personal strength | Visceral Adipose Tissue | No | 15.90 | 9.70 | - | - | - |  |  |
|  | Relating to others | Visceral Adipose Tissue | No | 11.30 | 4.70 | - | - | - |  |  |
|  | Spiritual change | Visceral Adipose Tissue | No | 15.60 | 7.20 | - | - | - |  |  |

Supplementary materials 5: Estimated marginal effects of PTG factors associated with cardiovascular risk outcomes not confirmed in regression models


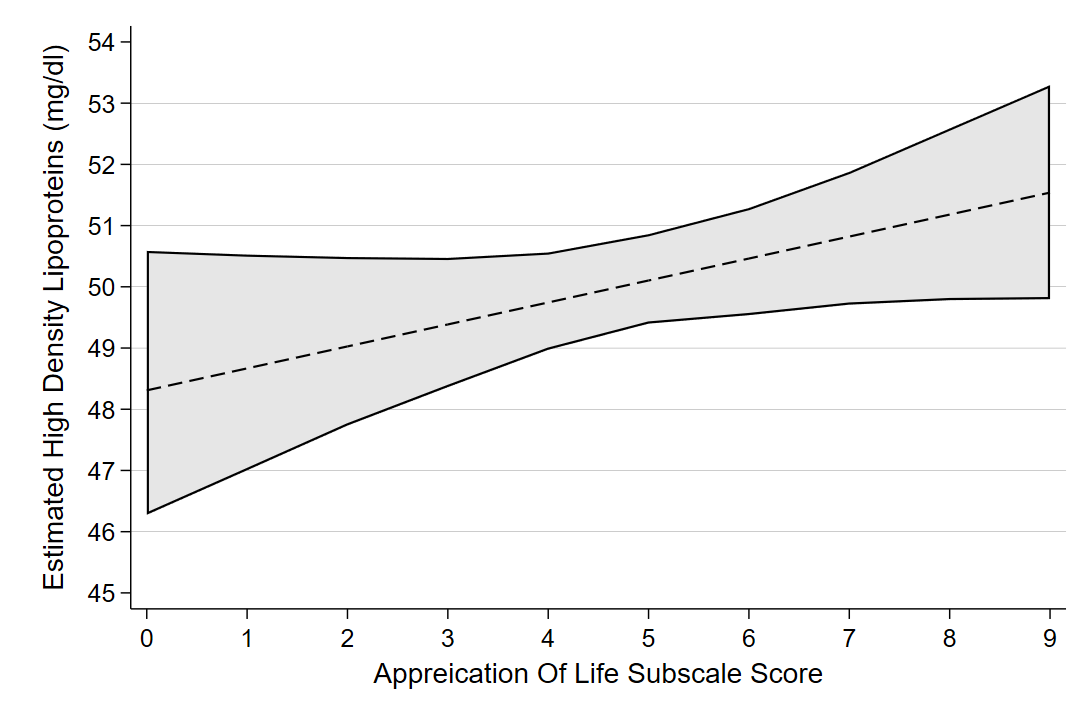


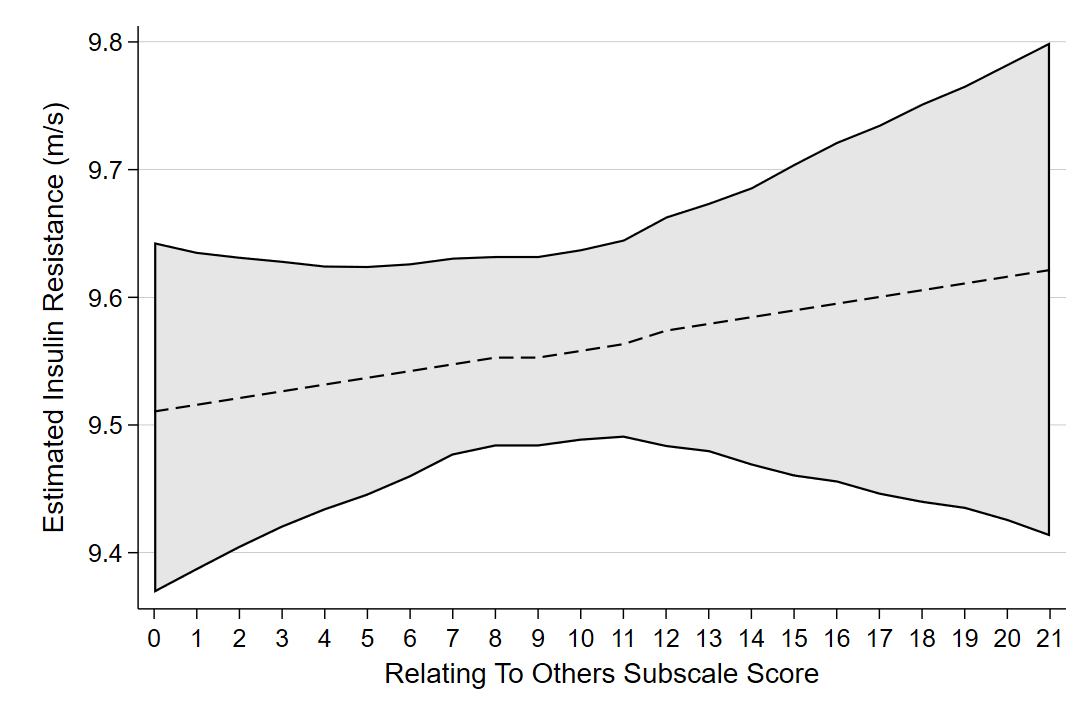


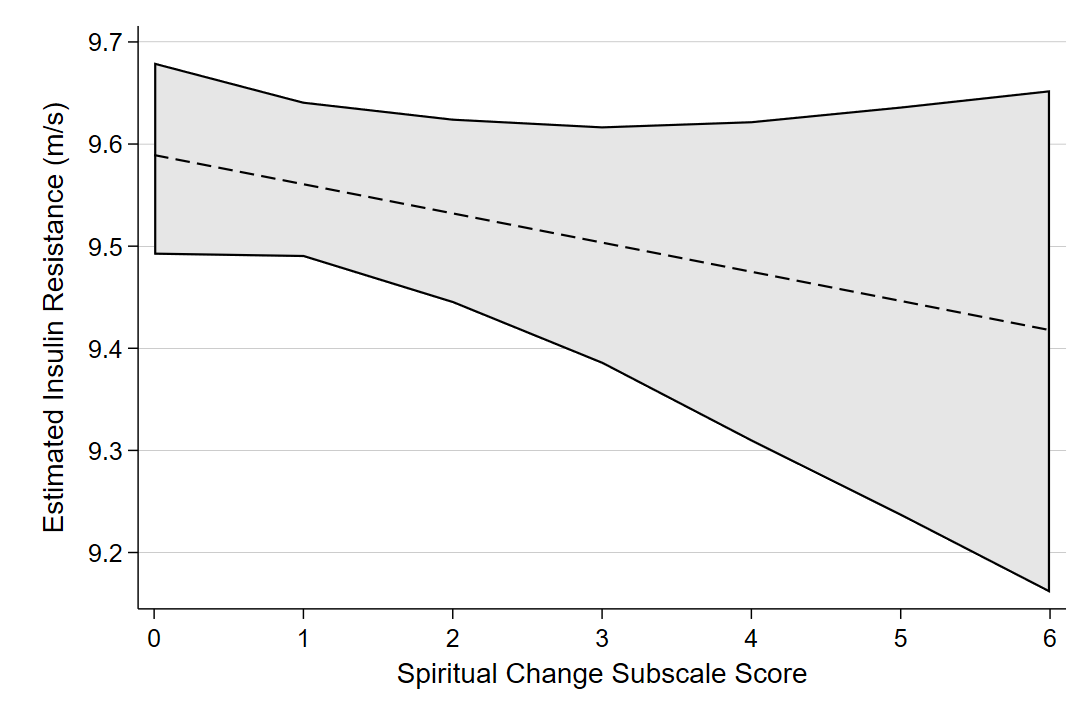


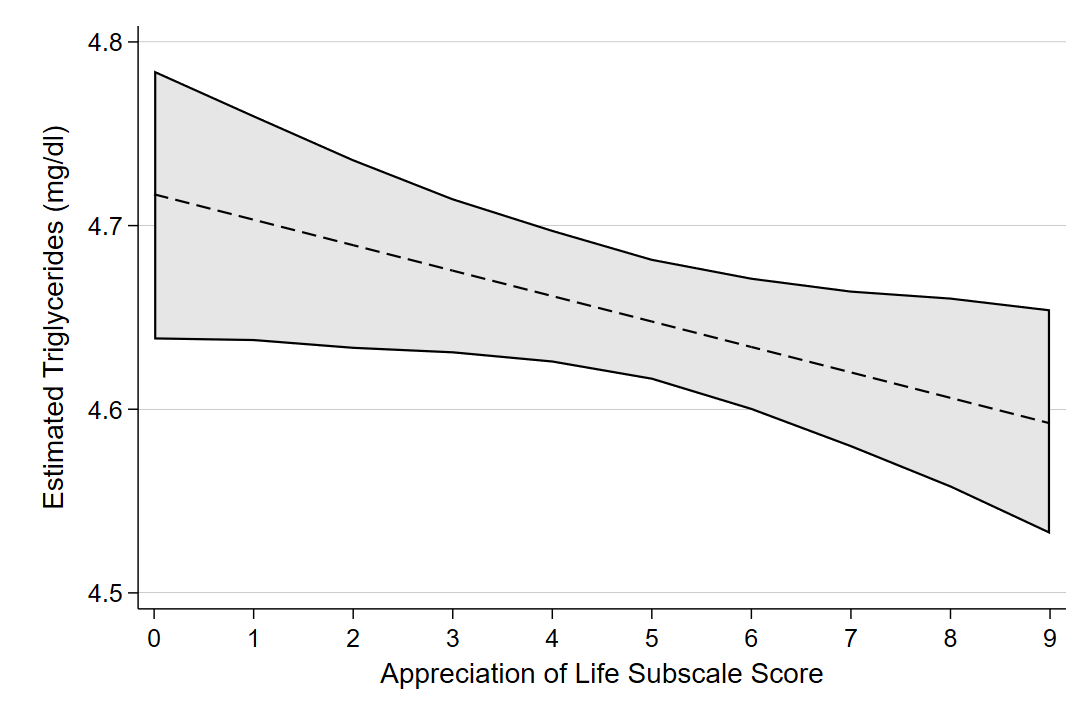


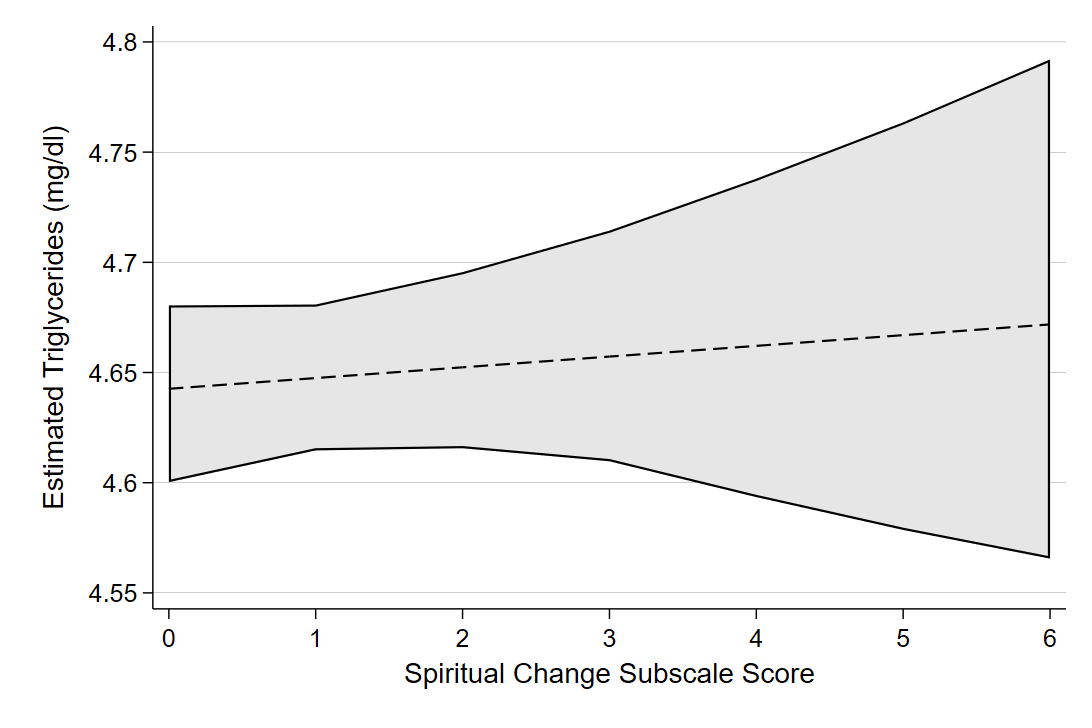

Supplement: sj-docx-1-hpq-10.1177_13591053241240196 – Supplemental material for The underlying mechanisms by which Post-Traumatic Growth is associated with cardiovascular health in male UK military personnel: The ADVANCE cohort study [file sj-docx-1-hpq-10.1177_13591053241240196.docx]
